# SIRT6 protects against endothelial dysfunction and atherosclerosis in mice

**DOI:** 10.18632/aging.100975

**Published:** 2016-05-30

**Authors:** Suowen Xu, Meimei Yin, Marina Koroleva, Michael A. Mastrangelo, Wenbo Zhang, Peter Bai, Peter J. Little, Zheng Gen Jin

**Affiliations:** ^1^ Aab Cardiovascular Research Institute, Department of Medicine, University of Rochester School of Medicine and Dentistry, Rochester, NY 14620, USA; ^2^ Department of Ophthalmology and Visual Sciences, University of Texas Medical Branch, Galveston, TX 77555, USA; ^3^ Department of Medical Chemistry, Faculty of Medicine, University of Debrecen, Debrecen, Hungary; ^4^ MTA-DE Lendület Laboratory of Cellular Metabolism Research Group, Debrecen, Hungary; ^5^ Research Center for Molecular Medicine, Faculty of Medicine, University of Debrecen, Debrecen, Hungary; ^6^ School of Pharmacy, The University of Queensland, Pharmacy Australia Centre of Excellence (PACE), Woolloongabba QLD 4102, Australia

**Keywords:** atherosclerosis, endothelial dysfunction, SIRT6, vascular inflammation, RNA-seq

## Abstract

SIRT6 is an important member of sirtuin family that represses inflammation, aging and DNA damage, three of which are causing factors for endothelial dysfunction. SIRT6 expression is decreased in atherosclerotic lesions from ApoE^−/−^ mice and human patients. However, the role of SIRT6 in regulating vascular endothelial function and atherosclerosis is not well understood. Here we show that SIRT6 protects against endothelial dysfunction and atherosclerosis. Global and endothelium-specific SIRT6 knockout mice exhibited impaired endothelium-dependent vasorelaxation. Moreover, SIRT6^+/−^ haploinsufficient mice fed a high-fat diet (HFD) also displayed impaired endothelium-dependent vasorelaxation. Importantly, SIRT6^+/−^;ApoE^−/−^ mice after HFD feeding exhibited exacerbated atherosclerotic lesion development, concurrent with increased expression of the proinflammatory cytokine VCAM-1. Loss- and gain-of-SIRT6 function studies in cultured human endothelial cells (ECs) showed that SIRT6 attenuated monocyte adhesion to ECs. RNA-sequencing profiling revealed that SIRT6 overexpression decreased the expression of multiple atherosclerosis-related genes, including proatherogenic gene TNFSF4 (tumor necrosis factor superfamily member 4). Chromatin immunoprecipitation assays showed that SIRT6 decreased TNFSF4 gene expression by binding to and deacetylating H3K9 at TNFSF4 gene promoter. Collectively, these findings demonstrate that SIRT6 play a pivotal role in maintaining endothelial function and increased SIRT6 activity could be a new therapeutic strategy to combat atherosclerotic disease.

## INTRODUCTION

Atherosclerosis is a chronic inflammatory disease that develops over decades leading to the clinical events of cardiovascular disease such as heart attacks and strokes. Atherosclerosis commences with the trapping of lipoproteins in the vessel wall by modified proteoglycans 1, 2] followed by disruption and dysfunction of the vascular endothelium and then a complex inflammatory reaction involving multiple immune cells some of which promote the development of the atherosclerotic plaques [3-5]. Plaques can be stable or labile where the latter are those that rupture to precipitate the life threatening clinical events [6]. Besides current cholesterol lowering therapy for the treatment of atherosclerosis, preferable therapies that address the disease process in the vessel wall are needed. One such potential strategy is to correct endothelial dysfunction, which is evidenced by impaired endothelium-dependent vasorelaxation, up-regulation of adhesion molecules and increased leukocyte adhesion and invasion, eventually resulting in the formation of a fatty streak, a prominent feature of atherosclerosis [7].

Sirtuins (SIRT1-SIRT7) belong to a family of NAD-dependent deacetylases that are involved in multiple cardiovascular pathologies [8-10]. Emerging evidence suggests that SIRT6, as a unique chromatin-associated deacetylase, share some of SIRT1-mediated protective effects [9, 11-15], such as inhibiting heart failure [16] and decelerating aging process [17] in mice. SIRT6 also halts endothelial inflammation [18], aging [19-21], as well as regulates dendritic cell differentiation, maturation and function [22]. In addition, SIRT6 expression is decreased in endothelial cells (ECs) under chronic stimulation with lipopolysaccharide (LPS) [18], hydrogen peroxide (H_2_O_2)_ [21] and high glucose [23-25], three of which are risk factors associated with endothelial dysfunction and atherogenesis. More recently, SIRT6 expression was reported to decrease in atherosclerotic ApoE^−/−^ mice [26, 27] and human patients [23, 27]. Moreover, SIRT6 genetic variants (rs107251, rs352493 and rs3760908) have been reported to associate with severity of coronary artery disease (CAD) [28, 29]. Therefore, further understanding of the atheroprotective role of SIRT6 would facilitate the translational exploitation of SIRT6 based therapeutic approaches in treating CAD.

In this report, we aim to address the role of SIRT6 in regulating endothelial dysfunction and atherosclerosis in mice and explore the underlying molecular mechanisms.

## RESULTS

### Global deletion of SIRT6 impairs endothelium-dependent vasorelaxation

We first characterized the expression of SIRT6 in murine tissues. We found that in normal C57BL/6J mice, SIRT6 gene was ubiquitously expressed in murine heart, lung, liver, kidney, aorta, skeletal muscle, spleen and brain (Figure S1). Noticeably, SIRT6 is highly expressed in EC-enriched tissues, such as lung, aorta and brain. The enrichment of SIRT6 in EC-containing tissues prompts us to investigate the role of SIRT6 in regulating endothelial function.

**Figure 1:**
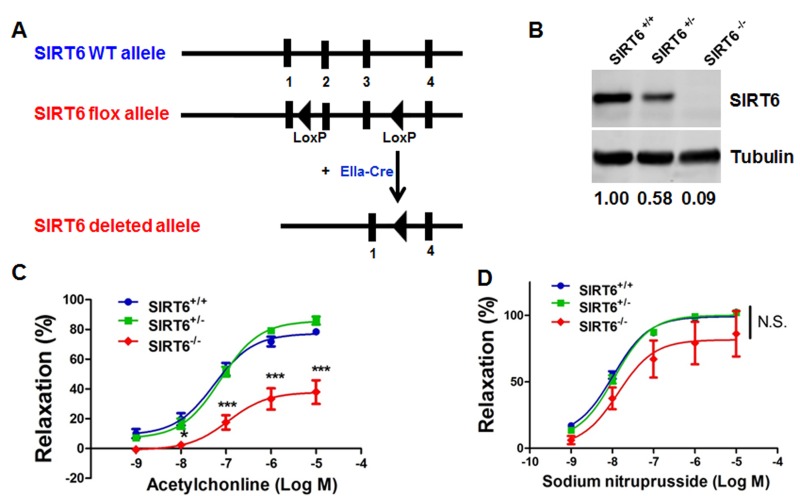
Global deletion of SIRT6 impairs endothelium-dependent relaxation (**A**) Schematic diagram of the transgenic mice used to generate SIRT6^−/−^ mice. (**B**) Western blot analysis showing SIRT6 protein expression in aortic lysates from SIRT6^+/+^, SIRT6^+/−^, and SIRT6^−/−^ mice, *n* = 4. (**C**) Vascular reactivity of WT (SIRT6^+/+^), SIRT6^+/−^ and SIRT6^−/−^ aortic rings to acetylcholine (Ach), ***P<0.001, compared to Sirt6+/+ littermates, *n* = 8-10; (**D**) Vascular reactivity of WT, SIRT6^+/−^ and SIRT6^−/−^ aortic rings to sodium nitroprusside (SNP), *n*=6-8.

To test whether SIRT6 deficiency in mice induces endothelial dysfunction, experiments were performed on SIRT6 global knock-out (SIRT6^−/−^) mice. These mice were obtained by crossing female *SIRT6^flox/flox^* mice [30] and male EIIa-Cre mice [31], which carry a Cre transgene under the control of the EIIa promoter that targets expression of Cre recombinase to the early mouse embryo and are useful for germ line deletion of loxP-flanked genes (Figure. 1A). Consistent with previous reports [11, 30], we found that SIRT6^−/−^ mice had lower body weight at 3-4 weeks of age (Figure S2) as well 12-weeks of age (Table S1) and most SIRT6^−/−^ mice die shortly after weaning. In survived SIRT6^−/−^ adult mice, we observed that, as compared to SIRT6^+/+^ littermates, SIRT6^−/−^ mice (Fig. S3A) had lower arterial systolic blood pressure and heart rate (Table S1), which may occur due to the complex phenotypes of SIRT6^−/−^ mice [11]. Western blot analysis showed that aortic SIRT6 protein was reduced by 42 % and 91% in SIRT6^+/−^ mice and SIRT6^−/−^ mice, respectively (Figure 1B). Endothelium-dependent vasorelaxation to acetylcholine (Ach) was significantly impaired in *SIRT6^−/−^* aortae compared to that in SIRT6^+/+^ aortae (Figure 1C). In contrast, endothelium-independent relaxation to sodium nitroprusside (SNP) did not differ significantly between SIRT6^+/+^ and *SIRT6^−/−^* aortae (Figure 1D). However, SIRT6 haploinsufficiency in mice (SIRT6^+/−^) shows similar Ach-induced endothelium-dependent vasorelaxation as well as SNP-induced endothelium-independent vasorelaxation under normal chow diet feeding conditions (Figure 1C and 1D).

### Endothelium-specific deletion of SIRT6 impairs endothelium-dependent vasorelaxation

**Figure 2:**
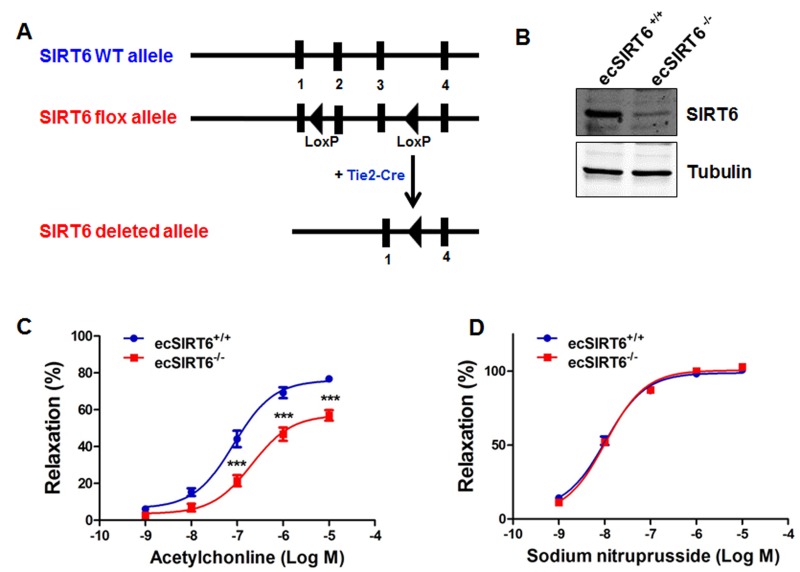
Endothelium-specific deletion of SIRT6 impairs endothelium-dependent relaxation (**A**) Schematic diagram of the transgenic mice used to generate endothelium-specific SIRT6 deficient (ecSIRT6^−/−^) mice and control SIRT6 wild-type (ecSIRT6^+/+^) mice. (**B**) Intimal endothelial cell lysates were harvested from ecSIRT6^+/+^ and ecSIRT6^−/−^ aortae and analyzed for the deletion of SIRT6 by Western blotting, *n*=4. (**C**) Reactivity of ecSIRT6^+/+^ and ecSIRT6^−/−^ aortic rings to acetylcholine (Ach), ***P<0.001, compared to ecSirt6+/+ littermates, *n* = 9; (**D**) Reactivity of ecSIRT6^+/+^ and ecSIRT6^−/−^ aortic rings to sodium nitroprusside (SNP), *n* = 10.

To ask whether endothelial SIRT6 regulates Ach-induced vasorelaxation, we generated endothelial cell-specific SIRT6 knockout (ecSIRT6^−/−^) mice and analyzed vasorelaxation of aortas from those mice. To this end, male Tie2-Cre mice [32] were cross-bred with *SIRT6^flox/flox^* [30] female mice to generate male Tie2-Cre; *SIRT6^flox/+^* mice, which were further intercrossed with *SIRT6^flox/flox^* females to obtain endothelium-specific SIRT6 knockout animals (ecSIRT6^−/−^, Figure 2A and Figure S3B). Analysis of the resulting genotypes revealed a close normal Mendelian frequency for ec*SIRT6^−/−^* mice (Table S2). The ec*SIRT6^−/−^* mice were viable and normal in size and did not display obvious physical, behavioral or reproductive abnormalities compared with littermate controls (SIRT6^flox/flox^). In addition, the ec*SIRT6^−/−^* mice were normotensive and had a normal heart rate (Table S1). To confirm the endothelium-specific SIRT6 deletion in these ec*SIRT6^−/−^* mice, Western blot analysis was performed. SIRT6 protein expression was significantly decreased in intimal EC lysate from ec*SIRT6^−/−^* aortae (Figure 2B). Subsequently, the effect of endothelial SIRT6 deletion on vascular reactivity was examined. We found that the concentration-dependent, Ach-induced vasorelaxation was significantly reduced in aortae of ecSIRT6^−/−^ mice compared with ecSIRT6^+/+^ (SIRT6^flox/flox^) control preparations (Figure 2C). By contrast, relaxations to SNP did not differ between ecSIRT6^+/+^ and ecSIRT6^−/−^ mice (Figure 2D). Taken together, these results indicate that endothelial SIRT6 is critical for the maintenance of normal endothelium-dependent vasorelaxation.

### SIRT6 haploinsufficiency impairs endothelium-dependent vasorelaxation in mice fed a high fat diet (HFD)

**Figure 3:**
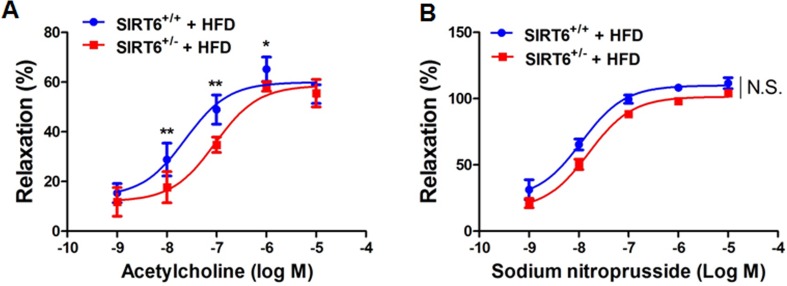
SIRT6 haploinsufficiency impairs endothelium-dependent vasorelaxation in mice fed high fat diet (**A**) Endothelium-dependent vasodilation was determined by relaxation of aortic rings pre-constricted with phenylephrine (PE, 10^−6^ mol/L). The dose-response curves of aortic rings to the vasodilator acetylcholine (Ach, 10^−9^−10^−5^ mol/L) in SIRT6^+/−^ mice and WT littermates under high fat diet (HFD) conditions for 12 weeks. (**B**) The dose-response curves of aortic rings to NO donor sodium nitroprusside (SNP, 10^−9^−10^−5^ mol/L) in SIRT6^+/−^ mice and WT littermates fed a HFD for 12 weeks. *P<0.05, **P<0.01, compared to WT littermates, *n*=5-6.

To further investigate whether SIRT6 haploin-sufficiency aggravates endothelial dysfunction under HFD feeding conditions, we challenged SIRT6^+/−^ mice and WT mice with a HFD for 3 months. We found that Ach-induced, endothelium-dependent vasodilation was significantly impaired in SIRT6^+/−^ mice, compared with SIRT6^+/+^ littermates (Figure 3A). However, the relaxation of the aortic rings in response to SNP in both genotypes of mice was similar (Figure 3B). These results indicate that SIRT6 haploinsufficiency aggravates endothelial dysfunction *in vivo* under hyper-cholesterolaemic dietary conditions.

### SIRT6 haploinsufficiency exacerbates the development of atherosclerosis in ApoE^−/−^ mice

**Figure 4:**
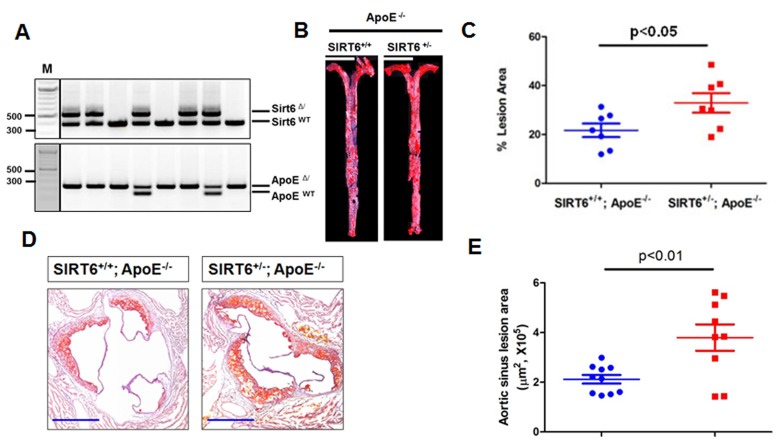
SIRT6 haploinsufficiency exacerbates atherogenesis in ApoE^−/−^ mice (**A**) Genotyping of SIRT6 and ApoE wild-type and mutant mice. SIRT6^+/−^/ApoE^−/−^ mice were generated by cross-breeding male SIRT6^+/−^/ApoE^+/−^ mice with ApoE^−/−^ females. Representative images show the results of genotypes by tail genomic DNA PCR method. SIRT6^−(Δ)^, and SIRT6^+(WT)^ bands appear at 524 bp and 390 bp, respectively. ApoE^−(Δ)^, ApoE^+(WT)^ bands appear at 245 bp and 155 bp, respectively. (**B**) SIRT6^+/−^/ApoE^−/−^ developed more atherosclerotic plaques in *en face* aorta. SIRT6^+/−^/ApoE^−/−^ and SIRT6^+/+^/ApoE^−/−^ mice were fed HFD for 8 weeks. The mice were then sacrificed and aortas from the aortic arch to the iliac arteries were examined for atherosclerotic lesion formation using the *en face* Oil-Red O staining. The images presented were a composite of 4-6 images captured at different regions of the same aorta, scale bar=4 mm. (**C**) Percentage of atherosclerotic lesions in the whole aorta was quantified by Image-Pro Plus software, *n*=7. (**D-E**) SIRT6^+/−^/ApoE^−/−^ developed more atherosclerotic lesions in the aortic sinus, *n*=9-10, scale bar=0.4 mm.

Impaired endothelium-dependent vasorelaxation is a hallmark of early phase of atherosclerosis. Thus, we hypothesized that SIRT6 protects against the development of atherosclerosis in mice. To test this hypothesis, SIRT6^+/−^ mice were bred with ApoE^−/−^ mice to obtain SIRT6^+/−^; ApoE^−/−^ mice (Figure 4A). Male SIRT6^+/−^; ApoE^−/−^ mice and their littermate controls male SIRT6^+/+^; ApoE^−/−^ mice at 8-weeks of age were put on a HFD for 8 additional weeks to accelerate atherosclerosis development. At the end of HFD feeding, mice were euthanized, and atherosclerosis lesion formation in aorta and aortic sinus were analyzed by *en face* analysis of the whole aorta and by cross-sectional analysis of the aortic sinus. Our data showed that SIRT6^+/−^;ApoE^−/−^ mice had more atherosclerotic plaques in the aorta (Figure 4B and 4C, *P* < 0.05) and the aortic sinus (Figure 4D and 4E, *P* < 0.01) than those in SIRT6^+/+^; ApoE^−/−^ control mice. Analysis of lipid profile suggests that SIRT6 haploinsufficiency significantly increased serum HDL levels without affecting TG and LDL/VLDL levels (Table S3).

### SIRT6 haploinsufficiency increases the expression of adhesion molecule VCAM-1 in ApoE^−/−^ mice

**Figure 5:**
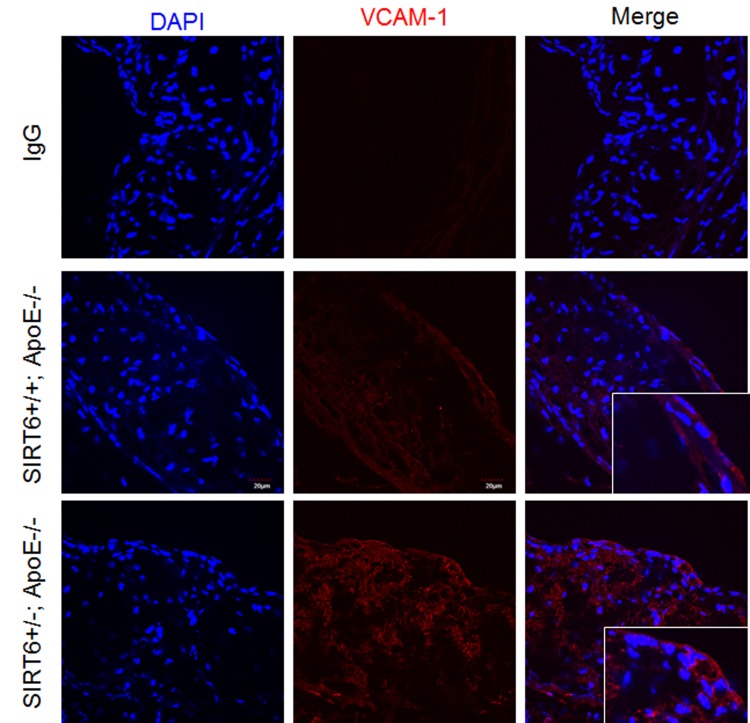
SIRT6 deficiency increases VCAM-1 expression in ApoE^−/−^ mice Aortic sinus from SIRT6^+/+^; ApoE^−/−^ and SIRT6^+/−^; ApoE^−/−^ mice were stained with VCAM-1 (red) antibody. DAPI was used to counterstain cell nuclei, bar=30 um, *n*=4-6/each genotype.

Endothelial cell adhesion molecules, such as vascular cell adhesion molecule-1 (VCAM-1), play an important role in atherogenesis by promoting monocyte adhesion to inflamed endothelium [7, 45]. Thus we examined the effect of SIRT6 deletion on VCAM-1 expression in aortic sinus. We observed that VCAM-1 was increased in atherosclerotic plaques of SIRT6^+/−^; ApoE^−/−^ compared with SIRT6^+/+^; ApoE^−/−^ mice (Figure 5). These results indicate that SIRT6 prevents adhesion molecule VCAM-1 expression.

### SIRT6 inhibits monocyte adhesion to endothelial cells

**Figure 6:**
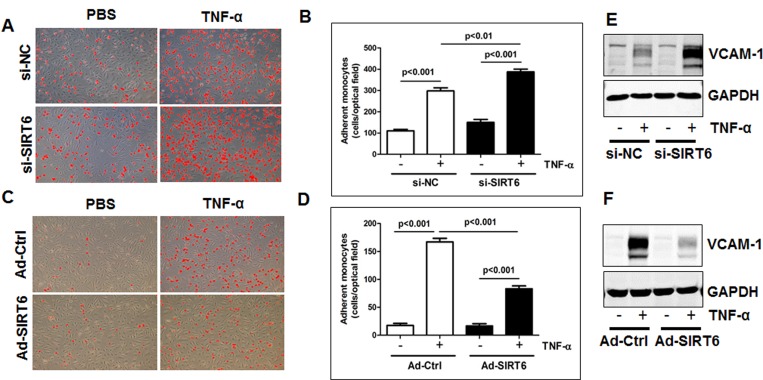
SIRT6 inhibits monocyte adhesion to endothelial cells by attenuating VCAM-1 (**A-B**) HUVECs transfected with SIRT6 siRNA (48 h, **A**) or infected with SIRT6 adenovirus (10 M.O.I., 24 h, **B**), and then monocyte adhesion to endothelial cells was determined. The representative images were shown after Image J adjustment of the threshold to label adhered monocytes with red color, original magnification, ×10. (**C-D**) Quantification of panel A and B, *n*=3-4. (**E-F**) HUVECs transfected with SIRT6 siRNA (48 h, **E**) or infected with SIRT6 adenovirus (10 M.O.I., 24 h, **F**) as indicated, then stimulated with TNF-α for 6 h, whole cell lysates were collected for Western blot analysis to detect VCAM-1 expression. GAPDH was used as the loading control, *n*=3.

To further understand the molecular mechanisms of SIRT6-mediated atheroprotection, we evaluated the effect of loss- and gain-of SIRT6 function on tumor necrosis factor alpha (TNF-α)-induced monocyte adhesion to ECs. HUVECs were transfected with SIRT6 siRNA for 48 h, or infected with SIRT6 adenovirus (the multiplicity of infection or M.O.I. 10) for 24 h (Figure S4). We then evaluated the effect of SIRT6 depletion and overexpression on monocyte adhesion to ECs. As shown in Figure 6A-D, the number of adhering human THP-1 monocytes to HUVECs was significantly increased (by 30%, *P*<0.01) by SIRT6 siRNA treatment and decreased (by 50%, *P*<0.001) by SIRT6 overexpression. Further studies of the mechanisms whereby SIRT6 inhibits monocyte adhesion showed that SIRT6 inhibited monocyte adhesion by decreasing TNF-α-induced expression of VCAM-1 in ECs (Figure 6E-F).

### SIRT6 overexpression alters endothelial transcriptional profile

To gain mechanistic insights into the effects of SIRT6 on endothelial gene transcriptome, we performed RNA-sequencing (RNA-seq) analysis of control adenovirus and SIRT6 adenovirus infected HUVECs. Gene ontology (GO), pathway enrichment and functional annotation clustering analysis were performed to classify differentially expressed genes and to identify the most significantly enriched GO terms/pathways/gene clusters. RNA-seq data showed that SIRT6 overexpression in HUVECs significantly downegulated the expression of 198 genes by >40% (Table S5).

**Figure 7:**
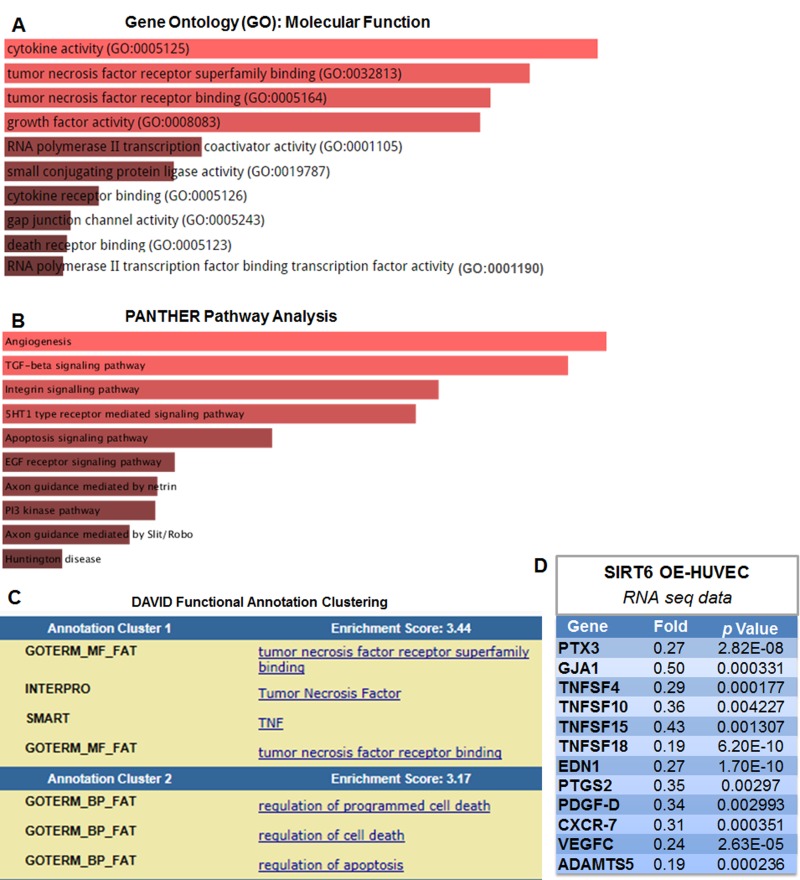
SIRT6 overexpression suppressed the expression of multiple atherosclerosis-related genes in human endothelial cells (**A-B**) Molecular Function Gene Ontology (GO) and pathway enrichment analysis of the 198 genes that were downregulated by SIRT6 overexpression, determined by RNA-seq, *n*=2/each condition. The lists of genes were analyzed using Enrichr database, based on the combined score ranking. The brighter the color, the more significantly enriched GO term and pathway displayed. Please refer to Table S4 and S5 for complete list of gene change. (**C**) DAVID functional annotation clustering analysis of genes downregulated by SIRT6 overexpression. (**D**) Selected atherosclerosis-related genes downregulated by SIRT6 overexpression (OE) detected by RNA-seq analysis.

Overrepresentation analysis of these genes using Enrichr database [33] revealed that the top three significantly overrepresented molecular function GO terms were cytokine and TNF-α receptor (superfamily) binding (Figure 7A). PANTHER pathway analysis suggests that SIRT6 modulates several important pathways in ECs, including angiogenesis, TGF-β signaling and integrin signaling (Figure 7B). To investigate the enrichment of functional annotation, we used Database for Annotation, Visualization and Integrated Discovery (DAVID) [34] to examine the 198 genes downregulated by SIRT6 overexpression. The functional annotation clustering results suggests that the top two DAVID-defined clusters which displayed enrichment scores (ES) >3 under high classification stringency parameters were: TNF-α receptor (super-family) binding and cell death (Figure 7C). Detailed analysis of RNA-seq data indicated that SIRT6 overexpression decreased the expression of multiple genes involved in vascular inflammation and atherosclerosis, including PTX3 (Pentraxin 3) [35], GJA1 (gap junction A1, also known as Connexin 43) [36], and TNFSF4 (tumor necrosis factor superfamily member 4, also known as OX40 ligand) [37] (Figure 7D and Table S4). In addition, SIRT6 overexpression upregulates several heat shock protein (HSP) genes that participate in DNA damage repair, including HSPA1A (Heat shock 70 kDa protein 1, also termed Hsp72), HSPA1B, and HSPA6 (Table S5).

### SIRT6 decreases atherosusceptible gene TNFSF4 by deacetylating H3K9 at gene promoter

**Figure 8:**
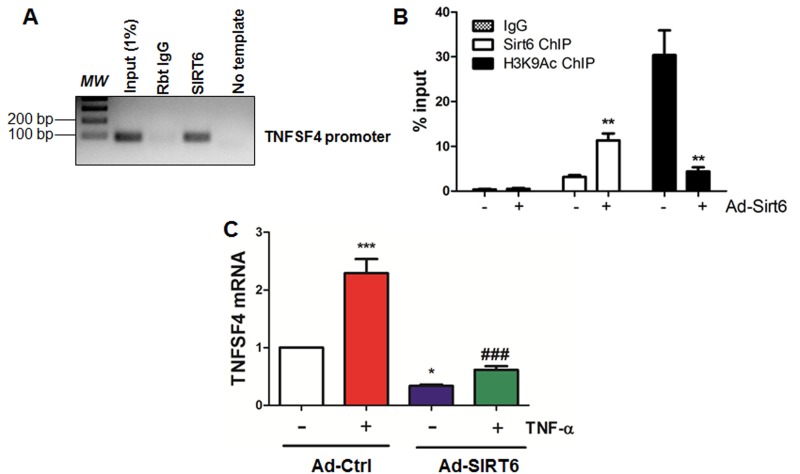
SIRT6 binds to and deacetylates H3K9 at TNFSF4 gene promoter (**A**) SIRT6 binds to TNFSF4 gene promoter in HUVECs. Sonicated chromatin from cultured HUVECs were used for ChIP assay, *n*=3. (**B**) SIRT6 deacetylates H3K9 at TNFSF4 gene promoter in HUVECs. HUVECs were infected with control adenovirus or SIRT6 adenovirus (M.O.I. 10) for 24 h. ChIP-qPCR was performed to determine SIRT6 and H3K9Ac occupancy at TNFSF4 gene proximal promoter. *n*=3, **P<0.01 versus Ad-ctrl. (**C**) SIRT6 inhibits basal and TNF-α induced TNFSF4 mRNA expression in HUVECs. HUVECs were infected with SIRT6 adenovirus (M.O.I 10) for 24 h before stimulation with TNF-α for 6 h, TNFSF4 mRNA was evaluated by q-RT-PCR after normalization with internal control, *n*=3, *P<0.05, ***P<0.001, compared to Ad-ctrl, ###*P*<0.001 compared to Ad-ctrl+TNF-α.

SIRT6 exerts its activity by deacetylating H3K9 [38, 39], H3K18 [40] and H3K56 [41, 42] at gene promoters. Particularly, SIRT6 attenuates NF-κB signaling by deacetylating H3K9 in the promoter region of NF-κB target genes including VCAM-1 [39]. To further validate whether TNFSF4, a critical atherosusceptible gene [37] associating with NF-κB pathway [43], is regulated by SIRT6, we performed chromatin immunoprecipitation (ChIP) assay. Our results indicate that SIRT6 binds to promoter region of TNFSF4 under basal conditions (Figure 8A), which was increased upon infection with SIRT6 adenovirus. However, the level of H3K9Ac binding to the TNFSF4 promoter region was significantly decreased by SIRT6 overexpression (Figure 8B). We next assessed mRNA expression of TNFSF4 in the presence or absence of TNF-α treatment. Real-time PCR analysis revealed that SIRT6 overexpression significantly reduced TNFSF4 expression under basal conditions and TNF-α stimulation (Figure 8C). These data suggest that SIRT6 decreases TNFSF4 expression by deacetylating H3K9 at TNFSF4 gene promoter.

## DISCUSSION

Atherosclerosis is a progressive, chronic inflammatory and immune disease, which underlies a high incidence of cardiovascular morbidity and mortality [7, 44-46]. Endothelial dysfunction followed by leukocyte infiltration leads to atherosclerotic plaque formation [7]. However the molecular mechanisms underlying endothelial dysfunction and atherogenesis remain incompletely understood. SIRT6 is a chromatin-associated histone deacetylase [12] highly expressed in vascular ECs from different vascular beds [18, 19], and SIRT6 represses endothelial inflammation [18] and aging [19-21]. However, the role of SIRT6 in regulating endothelial function and atherosclerosis remains poorly defined. The principal finding of the present study is that we demonstrate that SIRT6 reduces atherosclerotic lesion formation in a murine model of atherosclerosis through attenuating endothelial dysfunction and vascular inflammation.

Sirtuins are critical mediators of longevity induced by calorie restriction (CR) and CR-mimetic compounds such as resveratrol [9, 47]. Mounting evidence suggests that sirtuins regulates multiple cellular processes including lifespan extension, cellular metabolism and DNA damage repair [9, 47-50]. To date, SIRT1 is the best-characterized sirtuin in the cardiovascular system [8, 48, 49]. A large body of evidence shows that SIRT1 protects against atherosclerosis [48, 51-54] through multiple mechanisms, such as improving endothelial function [53, 55], inhibiting macrophage-derived foam cell formation [56], impeding the proliferation, migration [57] and DNA-damage associated apoptosis [58] of smooth muscle cells (SMCs), as well as suppressing thrombosis [59]. More recently, SIRT1 activating compound SRT3025 was shown to protect against atherosclerosis by modulating LDL-degrading enzyme proprotein convertase subtilisin/kexin type 9 (PCSK9) in LDLr^−/−^ mice [60]. However, there is limited information available regarding the role of other sirtuin family members in atherosclerosis. SIRT3 provides protection against cardiac hypertrophy, dyslipidaemia and cardiomyopathy [8], however, genetic ablation of SIRT3 have no impact on atherogenesis [61], suggesting distinctive effects of different sirtuins in atherosclerosis. In this study, we observed that global or endothelium-specific deficiency of SIRT6 impaired endothelium-dependent vasorelaxation. Because most of SIRT6 knockout mice die at 4 weeks of age [11], we used SIRT6 heterozygous (SIRT6^+/−^) mice to study whether SIRT6 haploin-sufficiency affects endothelium-dependent vasorelaxation and atherosclerosis. We observed that under normal diet conditions, no significant difference in vascular reactivity between aortas from SIRT6^+/−^ mice and wild type mice. However, under high-fat diet conditions, SIRT6 haploinsufficiency impaired endothelium-dependent vasorelaxation. We then generated SIRT6^+/−^; ApoE^−/−^ mice and fed them with HFD, and we found that those mice had increased atherosclerotic plaque formation. Mechanistically, SIRT6 diminishes monocyte adhesion by inhibiting the expression of adhesion molecules VCAM-1 in cultured ECs.

The development of atherosclerosis is a complex and multi-factorial process which involves the alteration of vascular endothelium hemostasis [62]. Many factors, such as impaired vasorelaxation, leukocyte adherence and extravasation, chronic inflammation, oxidative stress and aging cause endothelial dysfunction [62]. It has been shown that, in cultured ECs, SIRT6 negatively regulates EC aging [19, 21] and inflammation [18], suggesting that SIRT6 could be a vasculoprotective molecule in maintaining endothelial homeostasis. However, whether SIRT6 can improve vasorelaxation and inhibits monocyte adhesion remain unknown. Our gain- and loss-of-function studies in cultured ECs showed that SIRT6 inhibited TNF-α-induced monocyte adhesion to ECs. Two previous studies suggested that SIRT6 siRNA alone increases basal ICAM-1 expression [19, 26]. We observed that VCAM-1 expression were upregulated in SIRT6-depleted HUVECs in response to TNF-α stimulation. Conversely, TNF-α-induced VCAM-1 upregulation were mitigated by SIRT6 over-expression in ECs, suggesting that SIRT6 negatively regulates endothelial cell activation via suppressing the expression of adhesion molecules. The NF-κB transcriptional factor drives the expression of chemokines and adhesion molecules, such as ICAM-1 and VCAM-1, which recruit monocytes to diseased endothelium and initiates the development of atherosclerosis [63]. One possible explanation for the negative regulation of ICAM-1 and VCAM-1 by SIRT6 might be: SIRT6 deacetylates H3K9 at NF-κB target genes (ICAM-1 and VCAM-1) promoter and suppresses TNF-α-induced ICAM-1 and VCAM-1 expression [39]. Our RNA-seq data further provide a transcriptomic analysis of potential SIRT6 target genes. One candidate gene regulated by SIRT6 is TNFSF4, which is a NF-κB-associated atherosusceptible gene [37]. Our ChIP assays show that SIRT6 binds to TNFSF4 gene promoter and deacetylates H3K9 at TNFSF4 gene promoter, which results in SIRT6-dependent repression of TNFSF4 transcription in ECs. Whether similar mechanisms also control other potential SIRT6 target genes that we have identified in our RNA-seq experiments remain to be investigated in future studies.

Due to the fact that ApoE ^−/−^ mice fed a HFD show reduced SIRT6 expression [26, 27], it is of great importance to investigate in future studies whether EC-specific SIRT6 overexpression will rescue vascular inflammation and atherosclerosis development. Also, the model we used in this study is SIRT6 haploinsufficient mice, so the specific contributory roles of EC, SMC and macrophage derived SIRT6 in atherosclerosis development remains to be investigated using individual cell type-specific knockout mice in future studies.

In conclusion, our studies identify SIRT6 as a negative regulator of endothelial activation and atherosclerotic lesion development. Our findings also suggest that SIRT6-activating compounds could be potential therapeutics in the prevention/treatment of athero-sclerotic vascular diseases.

## METHODS

### Mice and diet

Animal procedures used in this study are in accord with institutional guidelines and were approved by the Institutional Animal Care and Use Committee of the University of Rochester Medical Center. Mice expressing Cre recombinase under control of the EIIa-Cre promoter/enhancer and SIRT6-floxed *(SIRT6^flox/flox^)* mice [30] were purchased from the Jackson Laboratory (Bar Harbor, ME, USA). LoxP recombination sites flank exons 2 and 3 of the *SIRT6* gene. To generate SIRT6 total knockout (*SIRT6^−/−^*) mice, *SIRT6^flox/flox^* mice were cross-bred with C57BL/6J EIIa-Cre mice. After that, SIRT6^+/−^ males were crossed to SIRT6^+/−^ females to obtain *SIRT6^−/−^* mice. After weaning, male SIRT6^+/+^, SIRT6^+/−^ and SIRT6^−/−^ mice were fed a normal chow diet or high fat diet (HFD) (TD.88137, Harlan Teklad) for 12 weeks before vascular reactivity analyses. We used both genders of mice for experiments involving whole-body SIRT6-deficient mice. To generate endothelium-specific SIRT6 knockout mice (Tie2-Cre*/SIRT6^flox/flox^*, defined as ec*SIRT6^−/−^*), female *SIRT6^flox/flox^* mice were cross-bred with male C57BL/6J Tie2-Cre mice. Then, male Tie2-Cre*/SIRT6^flox/+^* mice were further intercrossed with female SIRT6^flox/flox^ mice to obtain ec*SIRT6^−/−^* mice. Male SIRT6^flox/flox^ littermates were used as the control. To determine whether SIRT6 haploinsufficiency aggravates the development of atherosclerosis in ApoE^−/−^ mice, SIRT6^+/−^ males were crossed to ApoE^−/−^ females to obtain SIRT6^+/−^; ApoE^−/−^ mice. Male SIRT6^+/+^; ApoE^−/−^ littermates were used as the control. To accelerate the development of atherosclerosis, 8 weeks old SIRT6^+/−^;ApoE^−/−^ and SIRT6^+/+^;ApoE^−/−^ mice were fed a HFD (TD.88137, Harlan Teklad) for 8 weeks before endpoint analysis.

### Genotyping of experimental mice

Mice carrying a SIRT6 floxed allele were genotyped by PCR using primers GF1 (5′-GCTAATGGGAACGAGACCAA-3′) and GR1 (5′-ACCCACCTCTCTCCCCTAAA-3′) [30]. This primer pair amplifies a 390-bp fragment from the wild-type SIRT6 gene and 444 bp from the floxed allele. The deletion (Δ) allele of SIRT6 is amplified using primers GF1 and GR3 (5′-GCGTCCACTTCTCTTTCCTG-3′) [30], which produces a fragment of 524 bp. The PCR profile was set as follows: 94°C, 5 min; 94°C, 20 sec; 60°C, 20 sec; 72°C, 1 min (35 cycles); 72°C, 5 min; 10°C forever. Tail DNA was subject to regular RT-PCR using 2X GoTaq Green Master Mix (Promega, Madison, WI, USA). Reaction products are separated in 1.5% agarose gel and visualized with Image Lab 5.1 software (Bio-rad).

### Arterial blood pressure and heart rate measurements

Systolic arterial blood pressure (SBP) and heart rate (HR) were measured using an automated 6-chamber non-invasive tail-cuff plethysmography (BP-2000, Visitech System, Apex, NC, USA) [64]. The mice were habituated to this procedure for 5 days of training before the actual experiments that were performed for 2 days. Recordings were averaged 20 consecutive readings per day after 10 preliminary recordings.

### Vascular reactivity experiment

Mouse thoracic aorta were dissected and mounted in a four-chamber Multi-wire Myograph System (DMT-610M, Arhus, Denmark) [64-66]. Vascular segments were dissected free of loose connective and peri-aortic adipose tissue under dissecting microscope (Olympus SZX7), and cut into rings of equal length (2 mm). The rest of aorta was used for Western blot analysis. Artery segments were maintained at 37°C in Krebs physiological saline solution (PSS) of the following composition (in mM): 118.3 NaCl, 4.7 KCl, 2.5 CaCl_2_, 1.2 MgSO_4_, 25 NaHCO_3_, 1.2 KH_2_PO_4_, and 5.5 D-Glucose. PSS was pre-warmed at 37°C and saturated with air balanced-5% CO_2_ to maintain a pH of 7.4. Arteries were subject to a wake-up protocol by stimulation two times with a 60 mM-K^+^ Krebs solution (K-PSS), in which NaCl was substituted with KCl of equal molar concentration, for 3 min each at 10-min intervals. Subsequently, aortic ring contraction was induced with phenylephrine (PE, Sigma, 10^−6^ mol/L), and aortic ring relaxation was induced with cumulative additions of the endothelium dependent vasodilator acetylcholine (Ach, Sigma, 10^−9^ to 10^−5^ mol/L) or NO donor sodium nitroprusside (SNP, Sigma, 10^−9^ to 10^−5^ mol/L). Vasodilatory responses were expressed as percent relaxation relative to PE-induced vascular tone, with 100% representing full relaxation to basal tension. Force was recorded via a PowerLab 4/30 system (AD Instruments Ltd., UK) and analyzed using LabChart 7.0 Acquisition System (AD Instruments Ltd., UK).

### Measurement of serum lipid profile

Mice were fasted overnight before blood collection from retro-orbital plexus as previously described [67]. Serum was prepared and total triglycerides (TG), HDL, and LDL/VLDL concentrations were measured using commercial colorimetric kits (#K622-100 for TG, #K613-100 for HDL and LDL/VLDL; BioVision, Milpitas, CA). For sample preparation, 2 μl undiluted serum were used for detecting TG level. To detect HDL and LDL/VLDL levels, 40 μl mouse serum were mixed with 40 μl 2 X Precipitation Buffer. The mixture was incubated 10 min at room temperature and then centrifuged at 2000 g using bench-top microcentrifuge for 10 min. The supernatant (HDL fraction) was transferred to new tubes. 10 μl was supernatant was used for measuring HDL levels. The precipitates are the LDL/VLDL fraction, after another spin at above conditions; trace amount of HDL supernatant was carefully removed. The resulting pellet was dissolved in 400 μl PBS. 4 μl was used to measure LDL/VLDL levels. The amount of serum used is optimized to ensure the final readings fall within the detection limit of the kits. Absorbance was measured at 590 nm using a Wallac VICTOR™ 1420 Microplate Reader (PerkinElmer, Waltham, MA).

### Cell culture

Human umbilical vein endothelial cells (HUVECs) were obtained from fresh umbilical cord veins from normal pregnancies with patients' informed consent [66, 68]. HUVECs were cultured in M200 medium supplemented with 5% fetal bovine serum (FBS), 1% penicillin/streptomycin, 1% L-glutamine, 10 U/mL heparin and 25 μg/mL endothelial cell growth supplement (ECGS). The cells were grown at 37°C in humidified 5% CO_2_ and used for experiments between passages three and five. Human monocyte-derived THP-1 cells (gifted by Y. Cai) were maintained in RPMI 1640 medium supplemented with 10% heat-inactivated FBS, 2 mM L-glutamine, 100 μg/mL of streptomycin and 100 U/mL of penicillin. All reagents for cell culture were from Thermo Fisher Scientific unless specified otherwise.

### RNAi experiments

HUVECs were transfected at 80% confluence with 20 nM SMARTpool ON-TARGET*plus* SIRT6 siRNA (siSIRT6, #L-013306-00-0005) (Dharmacon, Lafayette, CO) or with ON-TARGET*plus* Non-targeting Control Pool siRNA (siNC, D-001810-10-05) in Opti-MEM (Gibco, Grand Island, NY, USA), using Lipofectamine 2000 (Invitrogen) [68]. After 4 h of transfection, the medium was changed to fresh M200 complete media, and cells were maintained for 48 h before further experiments.

### Analysis of atherosclerotic lesions

*En face* aorta and aortic sinus sectioning were prepared for Oil Red O staining as previously described in detail [66, 67, 69]. For the *en face* analysis of the aorta, the Oil Red O stained aortas were photographed and captured with ProgRes Speed XT^core^5 CCD camera (JENOPTIK AG, Germany) mounted on a microscope (Leica S8AP0, Germany). The pictures presented were a composite of 4-6 images captured at different regions of the same aorta. The total aortic surface area and the lesion area were measured by computer-assisted morphometry using NIH ImageJ software (http://imagej.nih.gov). The ratio of the lesion area to the total area was calculated. Quantitative analysis of the total lesion area in aortic sinus was also performed with the NIH ImageJ software.

### Western blot analysis

Whole cell lysates were prepared from cultured cells and aorta as described previously [66, 68]. For Western blots, total cell lysates (15-20 μg) were separated by SDS-PAGE, transferred to nitrocellulose membrane (Pall, East Hills, NY) and were subsequently blocked in LI-COR blocking buffer (LI-COR Biosciences, Lincoln, NE) at room temperature for 1 h. Then the blots were incubated overnight at 4°C with appropriate primary antibodies listed in Table S6. Then after being washed 3 times with 1 X Tris buffered saline with 0.1% Tween-20 (TBST), membranes were incubated with IRDye® 680RD Goat anti-Mouse IgG (H+L) or IRDye® 800CW Goat anti-Rabbit IgG (H + L) (1:10,000 dilution in 1XTBST; LI-COR) at room temperature for 30 min. Images were visualized by using an Odyssey Infrared Imaging System (LI-COR).

### Real-time quantitative PCR (qRT-PCR)

Total RNA was extracted from cultured human ECs and indicated mouse tissues using an RNeasy Mini kit (Qiagen) and Trizol Lysis Reagent (Invitrogen), respectively [66]. For reverse transcription, total RNA was converted into first strand complementary DNA (cDNA) using a High-Capacity cDNA Reverse Transcription Kit (Applied Biosystems) following the manufacturer's instructions. Regular genotyping RT-PCR was performed using 2X GoTaq Green Master Mix (Promega). Reaction products were separated in 1% agarose gel and visualized with Image Lab 5.1 software (Bio-rad). Quantitative real-time PCR was then performed with a Bio-Rad CFX96 Touch Real-Time PCR Detection System or Bio-Rad iQ5 real-time PCR thermal cycler, using iQ SYBR Green Supermix (Bio-Rad) for relative mRNA quantification. The sequences of all the primers used were listed in Table S7. The comparative cycle threshold (Ct) method (2^−ΔΔCt^) [70] was used to determine the relative mRNA expression of target genes after normalization to housekeeping gene GAPDH or β-actin.

### *In vitro* monocyte adhesion assay

HUVECs were treated with human SIRT6 siRNA (for 48 h) or SIRT6 adenovirus [21.66] (for 24 h) before stimulation with mouse recombinant TNF-α (5 ng/mL, Roche, Indianapolis, IN) for 6 h, then THP-1 monocytes were added to monolayers of HUVECs and incubated for an additional 30 min. Non-adherent THP-1 cells were removed by washing three to five times with pre-warmed serum free M200 media. Attached cells were then observed by an inverted microscope Zeiss Axiovert 40C microscope (magnification: ×10; numeric aperture: 0.25; Carl Zeiss) using a Canon A640 digital camera [66]. The number of monocytes attached to ECs was manually calculated using Image Pro-Plus software Version 6.2 (Media Cybernetics, Rockville, MD). The images presented are shown after Image J adjustment of the threshold to label adhered monocytes with red.

### Chromatin immunoprecipitation (ChIP) assay

ChIP assays were performed using EZ-Magna ChIP^TM^ A/G ChIP kits (#17-10086, EMD Millipore), with slight modifications. After treatment, HUVECs (3∼4×10^5^ cell equivalents per IP) were fixed with 1% formaldehyde for 10 min at room temperature by directly adding 37% formaldehyde (#252549, Sigma-Aldrich) to the culture media to cross-link the DNA-protein complex. Glycine (10X) was added to each dish to quench unreacted formaldehyde and to terminate cross-linking reaction. Next, cells were washed three times with cold 1XPBS and harvested by scraping using 1X PBS with 1XProtease Inhibitor Cocktail II (PIC). After centrifugation at 800 g for 5 min at 4°C, cell pellets were resuspended in Cell Lysis Buffer with PIC and incubated on ice for 15 min with brief vortex every 5 min. At the end of incubation, cell lysate were passed through BD U-100 29^G1/2^ Insulin Syringes to facilitate the release of nuclei. Nuclear pellets were then obtained by centrifugation at 800 g for another 5 min at 4°C and resuspended in Nuclei Lysis Buffer with PIC. The resulting material is sonicated on crushed ice water to create chromatin fragments ranging 200∼1000 bp in size by using Diagenode Bioruptor® UCD-200 Sonication System (Denville, NJ) (settings: 2×10 cycles of 30 sec pulses with 30 sec rest in between pulses and power setting at “High”). Lysates were then centrifuged at 12,000 g for 10 min at 6°C to pellet the precipitated SDS, and incubate with 20 μl fully resuspended Protein A/G magnetic beads and ChIP-validated rabbit SIRT6 [27, 40] and H3K9Ac [40] antibodies at 4°C for 4 h or overnight with rotation. Then equal amounts of chromatin were incubated overnight with control rabbit IgG as negative control. The protein A/G magnetic beads bound chromatin was pelleted with 12-tube Magnetic Separation Rack (#14654, Cell Signaling), followed by washing with Low Salt Wash Buffer, High Salt Wash Buffer, LiCl Wash Buffer and TE buffer sequentially. Chromatin-associated proteins were then digested with 1 μl proteinase K (10 mg/ml) for 2 h at 62°C with gentle shaking and incubate at 95°C for 10 min. Chromatin DNA were then purified with spin columns provided with the kit. Additional DNA purification kits were from Qiagen (QIAquick PCR Purification Kit, # 28104, Valencia, CA). Finally, real-time PCR was performed by using ChIP primers (Table S7). The amplification efficiency of PCR primers is >90% as calculated from a standard curve generated from serially diluted genomic DNA template. PCR products are analyzed within the linear amplification phase of PCR. To control for variation between ChIP fractions, the amount of each promoter region of interest in the initial chromatin (Input DNA, 1% unless indicated) fraction was used for a normalization factor for the number of genome equivalents used in the analysis. Input DNA Ct was adjusted from 1% to 100% equivalent by subtracting a Ct value of 6.644 or Log_2_100. ChIP-PCR of human GAPDH promoter region was used as the positive control for H3K9Ac ChIP (Figure S5). Data were presented as a signal relative to the total input fraction (%input) using comparative cycle threshold (Ct) (2^−[Ct(IP)-Ct (input)^) method.

### RNA sequencing (RNA-seq)

RNA was extracted using an RNeasy Mini kit (Qiagen, Valencia, CA) per manufacturer's instructions. High-quality RNA samples (pre-assessed by Nanodrop 2000) were further processed in the Genomics Research Center of the University of Rochester. Briefly, RNA quality assessed with the Agilent Bioanalyzer (Agilent, Santa Clara, CA). The TruSeq RNA Sample Preparation Kit V2 (Illumina, San Diego, CA) was used for next generation sequencing library construction per manufacturer's protocols. mRNA was purified from 100 ng total RNA with oligo-dT magnetic beads and fragmented. First-strand cDNA synthesis was performed with random hexamer priming followed by second-strand cDNA synthesis. End repair and 3′ adenylation was then performed on the double stranded cDNA. Illumina adaptors were ligated to both ends of the cDNA, purified by gel electrophoresis and amplified with PCR primers specific to the adaptor sequences to generate amplicons of approximately 200-500 bp in size. The amplified libraries were hybridized to the Illumina single end flow cell and amplified using the cBot (Illumina, San Diego, CA) at a concentration of 8 pM per lane. Single end reads of 100 nt are generated for each sample and aligned to the organism specific reference genome. The sequencing was performed using the Illumina high-throughput HiSeqTM 2500.

### Gene ontology, pathway enrichment and functional annotation clustering analysis

Gene ontology (GO), pathway enrichment analysis was performed using the Enrichr tools available at the Enrichr Web site (http://amp.pharm.mssm.edu/Enrichr/index.html) [33]. Functional annotation clustering analysis was performed using the DAVID Bioinformatics Resources 6.7 (https://david.ncifcrf.gov/) according to published literature [34].

### Statistical analysis

Data are presented as means ± SEM unless otherwise indicated. Statistical analysis was performed using GraphPad Prism Software Version 5.02 (GraphPad software, La Jolla, CA). Results were evaluated by *t*-test or by one- or two-way analysis of variance (ANOVA) when appropriate. When multiple comparisons were made, a Bonferroni correction was performed for each test. A *P* value *P*<0.05 were considered to be statistically significant.

## SUPPLEMENTAL DATA



## References

[1] Tabas I, Williams KJ, Boren J (2007). Subendothelial lipoprotein retention as the initiating process in atherosclerosis: update and therapeutic implications. Circulation.

[2] Little PJ, Osman N, O'Brien KD (2008). Hyperelongated biglycan: the surreptitious initiator of atherosclerosis. Curr Opin Lipidol.

[3] Libby P (2002). Inflammation in atherosclerosis. Nature.

[4] Little PJ, Chait A, Bobik A (2011). Cellular and cytokine-based inflammatory processes as novel therapeutic targets for the prevention and treatment of atherosclerosis. Pharmacol Ther.

[5] Ross R (1999). Atherosclerosis--an inflammatory disease. N Engl J Med.

[6] Davies MJ (1996). Stability and instability: two faces of coronary atherosclerosis. The Paul Dudley White Lecture 1995. Circulation.

[7] Libby P, Ridker PM, Hansson GK (2011). Progress and challenges in translating the biology of atherosclerosis. Nature.

[8] Winnik S, Auwerx J, Sinclair DA, Matter CM (2015). Protective effects of sirtuins in cardiovascular diseases: from bench to bedside. Eur Heart J.

[9] Finkel T, Deng CX, Mostoslavsky R (2009). Recent progress in the biology and physiology of sirtuins. Nature.

[10] D'Onofrio N, Vitiello M, Casale R, Servillo L, Giovane A, Balestrieri ML (2015). Sirtuins in vascular diseases: Emerging roles and therapeutic potential. Biochim Biophys Acta.

[11] Mostoslavsky R, Chua KF, Lombard DB, Pang WW, Fischer MR, Gellon L, Liu P, Mostoslavsky G, Franco S, Murphy MM, Mills KD, Patel P, Hsu JT (2006). Genomic instability and aging-like phenotype in the absence of mammalian SIRT6. Cell.

[12] Kugel S, Mostoslavsky R (2014). Chromatin and beyond: the multitasking roles for SIRT6. Trends Biochem Sci.

[13] Houtkooper RH, Pirinen E, Auwerx J (2012). Sirtuins as regulators of metabolism and healthspan. Nat Rev Mol Cell Biol.

[14] Cohen HY, Miller C, Bitterman KJ, Wall NR, Hekking B, Kessler B, Howitz KT, Gorospe M, de Cabo R, Sinclair DA (2004). Calorie restriction promotes mammalian cell survival by inducing the SIRT1 deacetylase. Science.

[15] Lombard DB (2009). Sirtuins at the breaking point: SIRT6 in DNA repair. Aging (Albany NY).

[16] Sundaresan NR, Vasudevan P, Zhong L, Kim G, Samant S, Parekh V, Pillai VB, Ravindra PV, Gupta M, Jeevanandam V, Cunningham JM, Deng CX, Lombard DB (2012). The sirtuin SIRT6 blocks IGF-Akt signaling and development of cardiac hypertrophy by targeting c-Jun. Nat Med.

[17] Kanfi Y, Naiman S, Amir G, Peshti V, Zinman G, Nahum L, Bar-Joseph Z, Cohen HY (2012). The sirtuin SIRT6 regulates lifespan in male mice. Nature.

[18] Lappas M (2012). Anti-inflammatory properties of sirtuin 6 in human umbilical vein endothelial cells. Mediators Inflamm.

[19] Cardus A, Uryga AK, Walters G, Erusalimsky JD (2013). SIRT6 protects human endothelial cells from DNA damage, telomere dysfunction, and senescence. Cardiovasc Res.

[20] Shen J, Ma W, Liu Y (2013). Deacetylase SIRT6 deaccelerates endothelial senescence. Cardiovasc Res.

[21] Liu R, Liu H, Ha Y, Tilton RG, Zhang W (2014). Oxidative stress induces endothelial cell senescence via downregulation of Sirt6. Biomed Res Int.

[22] Lasiglie D, Boero S, Bauer I, Morando S, Damonte P, Cea M, Monacelli F, Odetti P, Ballestrero A, Uccelli A, Mostoslavsky R, Poggi A, Nencioni A (2016). Sirt6 regulates dendritic cell differentiation, maturation, and function. Aging (Albany NY).

[23] Balestrieri ML, Rizzo MR, Barbieri M, Paolisso P, D'Onofrio N, Giovane A, Siniscalchi M, Minicucci F, Sardu C, D'Andrea D, Mauro C, Ferraraccio F, Servillo L (2015). Sirtuin 6 expression and inflammatory activity in diabetic atherosclerotic plaques: effects of incretin treatment. Diabetes.

[24] Mortuza R, Chen S, Feng B, Sen S, Chakrabarti S (2013). High glucose induced alteration of SIRTs in endothelial cells causes rapid aging in a p300 and FOXO regulated pathway. PLoS One.

[25] D'Onofrio N, Servillo L, Giovane A, Casale R, Vitiello M, Marfella R, Paolisso G, Balestrieri ML (2016). Ergothioneine oxidation in the protection against high-glucose induced endothelial senescence: involvement of SIRT1 and SIRT6. Free Radic Biol Med.

[26] Liu Z, Wang J, Huang X, Li Z, Liu P (2016). Deletion of sirtuin 6 accelerates endothelial dysfunction and atherosclerosis in apolipoprotein E-deficient mice. Transl Res.

[27] Zhang ZQ, Ren SC, Tan Y, Li ZZ, Tang X, Wang TT, Hao DL, Zhao X, Chen HZ, Liu DP (2016). Epigenetic regulation of NKG2D ligands is involved in exacerbated atherosclerosis development in Sirt6 heterozygous mice. Sci Rep.

[28] Dong C, Della-Morte D, Wang L, Cabral D, Beecham A, McClendon MS, Luca CC, Blanton SH, Sacco RL, Rundek T (2011). Association of the sirtuin and mitochondrial uncoupling protein genes with carotid plaque. PLoS One.

[29] Tang SS, Xu S, Cheng J, Cai MY, Chen L, Liang LL, Yang XL, Chen C, Liu XG, Xiong XD (2016). Two tagSNPs rs352493 and rs3760908 within SIRT6 Gene Are Associated with the Severity of Coronary Artery Disease in a Chinese Han Population. Dis Markers.

[30] Xiao C, Kim HS, Lahusen T, Wang RH, Xu X, Gavrilova O, Jou W, Gius D, Deng CX (2010). SIRT6 deficiency results in severe hypoglycemia by enhancing both basal and insulin-stimulated glucose uptake in mice. J Biol Chem.

[31] Lakso M, Pichel JG, Gorman JR, Sauer B, Okamoto Y, Lee E, Alt FW, Westphal H (1996). Efficient in vivo manipulation of mouse genomic sequences at the zygote stage. Proc Natl Acad Sci U S A.

[32] Koni PA, Joshi SK, Temann UA, Olson D, Burkly L, Flavell RA (2001). Conditional vascular cell adhesion molecule 1 deletion in mice: impaired lymphocyte migration to bone marrow. J Exp Med.

[33] Chen EY, Tan CM, Kou Y, Duan Q, Wang Z, Meirelles GV, Clark NR, Ma'ayan A (2013). Enrichr: interactive and collaborative HTML5 gene list enrichment analysis tool. BMC Bioinformatics.

[34] Huang da W, Sherman BT, Lempicki RA (2009). Systematic and integrative analysis of large gene lists using DAVID bioinformatics resources. Nat Protoc.

[35] Shindo A, Tanemura H, Yata K, Hamada K, Shibata M, Umeda Y, Asakura F, Toma N, Sakaida H, Fujisawa T, Taki W, Tomimoto H (2014). Inflammatory biomarkers in atherosclerosis: pentraxin 3 can become a novel marker of plaque vulnerability. PLoS One.

[36] Chadjichristos CE, Matter CM, Roth I, Sutter E, Pelli G, Luscher TF, Chanson M, Kwak BR (2006). Reduced connexin43 expression limits neointima formation after balloon distension injury in hypercholesterolemic mice. Circulation.

[37] Wang X, Ria M, Kelmenson PM, Eriksson P, Higgins DC, Samnegard A, Petros C, Rollins J, Bennet AM, Wiman B, de Faire U, Wennberg C, Olsson PG (2005). Positional identification of TNFSF4, encoding OX40 ligand, as a gene that influences atherosclerosis susceptibility. Nat Genet.

[38] Michishita E, McCord RA, Berber E, Kioi M, Padilla-Nash H, Damian M, Cheung P, Kusumoto R, Kawahara TL, Barrett JC, Chang HY, Bohr VA, Ried T (2008). SIRT6 is a histone H3 lysine 9 deacetylase that modulates telomeric chromatin. Nature.

[39] Kawahara TL, Michishita E, Adler AS, Damian M, Berber E, Lin M, McCord RA, Ongaigui KC, Boxer LD, Chang HY, Chua KF (2009). SIRT6 links histone H3 lysine 9 deacetylation to NF-kappaB-dependent gene expression and organismal life span. Cell.

[40] Tasselli L, Xi Y, Zheng W, Tennen RI, Odrowaz Z, Simeoni F, Li W, Chua KF (2016). SIRT6 deacetylates H3K18ac at pericentric chromatin to prevent mitotic errors and cellular senescence. Nat Struct Mol Biol.

[41] Michishita E, McCord RA, Boxer LD, Barber MF, Hong T, Gozani O, Chua KF (2009). Cell cycle-dependent deacetylation of telomeric histone H3 lysine K56 by human SIRT6. Cell Cycle.

[42] Toiber D, Erdel F, Bouazoune K, Silberman DM, Zhong L, Mulligan P, Sebastian C, Cosentino C, Martinez-Pastor B, Giacosa S, D'Urso A, Naar AM, Kingston R (2013). SIRT6 recruits SNF2H to DNA break sites, preventing genomic instability through chromatin remodeling. Mol Cell.

[43] Godefroy E, Gallois A, Idoyaga J, Merad M, Tung N, Monu N, Saenger Y, Fu Y, Ravindran R, Pulendran B, Jotereau F, Trombetta S, Bhardwaj N (2014). Activation of toll-like receptor-2 by endogenous matrix metalloproteinase-2 modulates dendritic-cell-mediated inflammatory responses. Cell Rep.

[44] Hansson GK, Libby P (2006). The immune response in atherosclerosis: a double-edged sword. Nat Rev Immunol.

[45] Libby P, Ridker PM, Hansson GK (2009). Inflammation in atherosclerosis: from pathophysiology to practice. J Am Coll Cardiol.

[46] Xu S, Bai P, Little PJ, Liu P (2014). Poly(ADP-ribose) polymerase 1 in atherosclerosis: from molecular mechanisms to therapeutic implications. Med Res Rev.

[47] Baur JA, Ungvari Z, Minor RK, Le Couteur DG, de Cabo R (2012). Are sirtuins viable targets for improving healthspan and lifespan?. Nat Rev Drug Discov.

[48] Winnik S, Stein S, Matter CM (2012). SIRT1 - an anti-inflammatory pathway at the crossroads between metabolic disease and atherosclerosis. Curr Vasc Pharmacol.

[49] Yang Z, Ming XF (2010). The vascular SIRTainty. Aging (Albany NY).

[50] McCord RA, Michishita E, Hong T, Berber E, Boxer LD, Kusumoto R, Guan S, Shi X, Gozani O, Burlingame AL, Bohr VA, Chua KF (2009). SIRT6 stabilizes DNA-dependent protein kinase at chromatin for DNA double-strand break repair. Aging (Albany NY).

[51] Stein S, Matter CM (2011). Protective roles of SIRT1 in atherosclerosis. Cell Cycle.

[52] Chen Z, Peng IC, Cui X, Li YS, Chien S, Shyy JY (2010). Shear stress, SIRT1, and vascular homeostasis. Proc Natl Acad Sci U S A.

[53] Zhang QJ, Wang Z, Chen HZ, Zhou S, Zheng W, Liu G, Wei YS, Cai H, Liu DP, Liang CC (2008). Endothelium-specific overexpression of class III deacetylase SIRT1 decreases atherosclerosis in apolipoprotein E-deficient mice. Cardiovasc Res.

[54] Bai B, Liang Y, Xu C, Lee MY, Xu A, Wu D, Vanhoutte PM, Wang Y (2012). Cyclin-dependent kinase 5-mediated hyperphosphorylation of sirtuin-1 contributes to the development of endothelial senescence and atherosclerosis. Circulation.

[55] Stein S, Schafer N, Breitenstein A, Besler C, Winnik S, Lohmann C, Heinrich K, Brokopp CE, Handschin C, Landmesser U, Tanner FC, Luscher TF, Matter CM (2010). SIRT1 reduces endothelial activation without affecting vascular function in ApoE−/− mice. Aging (Albany NY).

[56] Stein S, Lohmann C, Schafer N, Hofmann J, Rohrer L, Besler C, Rothgiesser KM, Becher B, Hottiger MO, Boren J, McBurney MW, Landmesser U, Luscher TF (2010). SIRT1 decreases Lox-1-mediated foam cell formation in atherogenesis. Eur Heart J.

[57] Li L, Zhang HN, Chen HZ, Gao P, Zhu LH, Li HL, Lv X, Zhang QJ, Zhang R, Wang Z, She ZG, Zhang R, Wei YS (2011). SIRT1 acts as a modulator of neointima formation following vascular injury in mice. Circ Res.

[58] Gorenne I, Kumar S, Gray K, Figg N, Yu H, Mercer J, Bennett M (2013). Vascular smooth muscle cell sirtuin 1 protects against DNA damage and inhibits atherosclerosis. Circulation.

[59] Breitenstein A, Stein S, Holy EW, Camici GG, Lohmann C, Akhmedov A, Spescha R, Elliott PJ, Westphal CH, Matter CM, Luscher TF, Tanner FC (2011). Sirt1 inhibition promotes in vivo arterial thrombosis and tissue factor expression in stimulated cells. Cardiovasc Res.

[60] Miranda MX, van Tits LJ, Lohmann C, Arsiwala T, Winnik S, Tailleux A, Stein S, Gomes AP, Suri V, Ellis JL, Lutz TA, Hottiger MO, Sinclair DA (2015). The Sirt1 activator SRT3025 provides atheroprotection in Apoe−/− mice by reducing hepatic Pcsk9 secretion and enhancing Ldlr expression. Eur Heart J.

[61] Winnik S, Gaul DS, Preitner F, Lohmann C, Weber J, Miranda MX, Liu Y, van Tits LJ, Mateos JM, Brokopp CE, Auwerx J, Thorens B, Luscher TF (2014). Deletion of Sirt3 does not affect atherosclerosis but accelerates weight gain and impairs rapid metabolic adaptation in LDL receptor knockout mice: implications for cardiovascular risk factor development. Basic Res Cardiol.

[62] Verma S, Buchanan MR, Anderson TJ (2003). Endothelial function testing as a biomarker of vascular disease. Circulation.

[63] Xu S, Ogura S, Chen J, Little PJ, Moss J, Liu P (2013). LOX-1 in atherosclerosis: biological functions and pharmacological modifiers. Cell Mol Life Sci.

[64] Korshunov VA, Daul M, Massett MP, Berk BC (2007). Axl mediates vascular remodeling induced by deoxycorticosterone acetate-salt hypertension. Hypertension.

[65] Zhang DX, Mendoza SA, Bubolz AH, Mizuno A, Ge ZD, Li R, Warltier DC, Suzuki M, Gutterman DD (2009). Transient receptor potential vanilloid type 4-deficient mice exhibit impaired endothelium-dependent relaxation induced by acetylcholine in vitro and in vivo. Hypertension.

[66] Xu S, Liu B, Yin M, Koroleva M, Mastrangelo M, Ture S, Morrell C, Zhang DX, Fisher EA, Jin ZG (2016). A novel TRPV4-specific agonist inhibits monocyte adhesion and atherosclerosis. Oncotarget.

[67] Xu S, Little PJ, Lan T, Huang Y, Le K, Wu X, Shen X, Huang H, Cai Y, Tang F, Wang H, Liu P (2011). Tanshinone II-A attenuates and stabilizes atherosclerotic plaques in apolipoprotein-E knockout mice fed a high cholesterol diet. Arch Biochem Biophys.

[68] Xu S, Ha CH, Wang W, Xu X, Yin M, Jin FQ, Mastrangelo M, Koroleva M, Fujiwara K, Jin ZG (2016). PECAM1 regulates flow-mediated Gab1 tyrosine phosphorylation and signaling. Cell Signal.

[69] Xu S, Huang Y, Xie Y, Lan T, Le K, Chen J, Chen S, Gao S, Xu X, Shen X, Huang H, Liu P (2010). Evaluation of foam cell formation in cultured macrophages: an improved method with Oil Red O staining and DiI-oxLDL uptake. Cytotechnology.

[70] Xu S, Liu Z, Huang Y, Le K, Tang F, Huang H, Ogura S, Little PJ, Shen X, Liu P (2012). Tanshinone II-A inhibits oxidized LDL-induced LOX-1 expression in macrophages by reducing intracellular superoxide radical generation and NF-kappaB activation. Transl Res.

