# Supplementary material for: SIRT6 protects against endothelial dysfunction and atherosclerosis in mice
Source: Aging (Albany NY). 2016 May 30;8(5):1064–78. doi: 10.18632/aging.100975 (PMC4931854; doi:10.18632/aging.100975)
Supplement: Supplementary file 1 [file aging-08-1064-s001.pdf]

SUPPLEMENTAL DATA

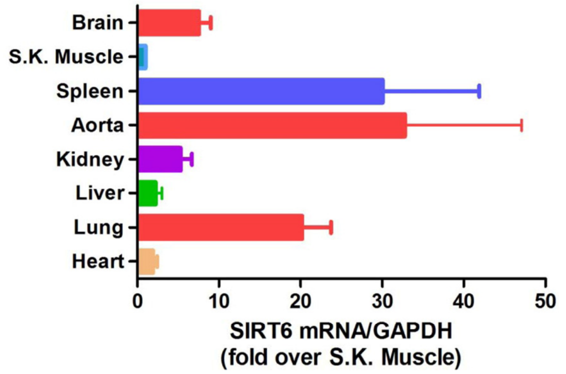

Figure S1. Tissue distribution of SIRT6. SIRT6 is expressed in multiple mouse tissues (C57BL/6J mice, 3 month old) as measured by qRT-PCR. In addition to the spleen (which harbors erythrocytes, monocytes, B- and T-lymphocytes), endothelial cell-enriched aortic, lung and brain tissues show the enrichment of SIRT6 gene expression. Relative SIRT6 mRNA to GAPDH level in skeletal (S.K.) muscle was set at 1.0, *n* = 3.

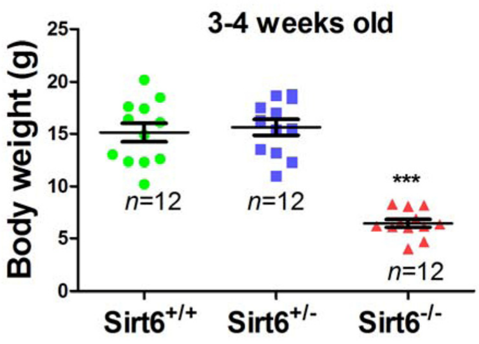

Figure S2. Body weight of SIRT6 wild type and mutant mice at 3-4 weeks of age. Data are presented as Mean±SEM, ***P<0.001.

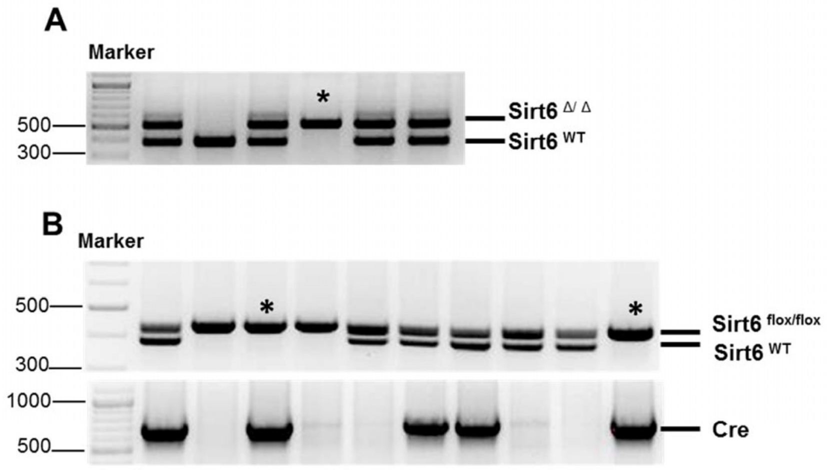

Figure S3. Genotyping of total and endothelium-specific knockout mice. SIRT6$^{-/-}$ mice (*, panel **A**) were generated by SIRT6$^{+/-}$ cross-breeding. Endothelium-specific SIRT6 knockout mice (ecSIRT6$^{-/-}$, labelled as *, Tie2-Cre; SIRT6 $^{flox/flox}$, panel **B**) were generated by cross-breeding male Tie2-Cre; SIRT6 $^{flox/+}$ mice with female SIRT6 flox/flox mice. Representative image shows the results of genotypes by tail genomic DNA PCR method. SIRT6$^{\Delta}$, SIRT6^{flox} and SIRT6^{WT} band appears at 524 bp, 444 bp, and 390 bp, respectively.

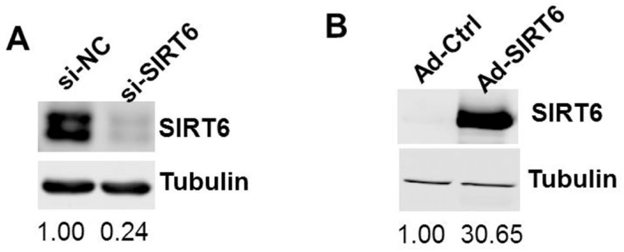

Figure S4. Silencing efficiency of SIRT6 siRNA and over-expression efficiency of SIRT6 adenovirus. Western blot analysis of protein expression of SIRT6 in HUVECs transfected with SIRT6 siRNA (20 nM, 48 h, **A**) or infected with SIRT6 adenovirus (M.O.I. 10, 24 h, **B**), *n*=4 for panel **A** and *n*=3 for panel **B**.

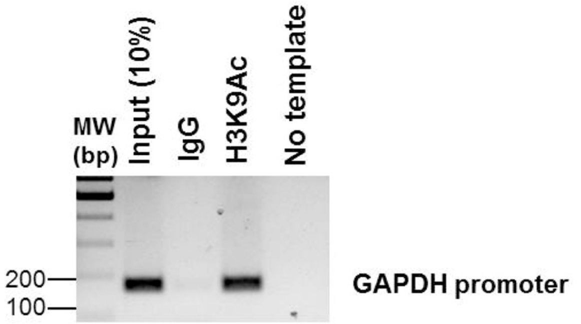

Figure S5. Validation of H3K9 antibody for ChIP assay. ChIP was performed using chromatin from HUVECs and incubated with either rabbit H3K9Ac or normal rabbit IgG as the immunoprecipitating antibody. Purified DNA was then analyzed by PCR using control primers specific for the GAPDH promoter (166 bp). PCR product was observed in the anti-H3K9Ac ChIP (lane 3) but not in the normal rabbit IgG ChIP (lane 2). GAPDH promoter specific DNA was also observed in the Input (lane 1) and not in the "No template" PCR control (lane 4). Representative blots from 3 independent experiments.

Supplementary Table S1. Body weight, systolic blood pressure and heart rate of SIRT6 total knockout (SIRT6$^{-/-}$) and endothelium-specific knockout (ecSIRT6$^{-/-}$, Tie2-Cre; SIRT6 $^{flox/flox}$) mice. Data are presented as Mean±SD, n=8-15. **P*<0.05; ****P*<0.001.

Genotypes	Body weight (g)	Systolic blood pressure (mmHg)	Heart rate (bpm)
SIRT6 $^{+/+}$	35.8±6.2	110.8±9.0	647.5±53.4
SIRT6 $^{+/-}$	35.7±4.9	118.3±11.9	663.8±49.7
SIRT6 $^{-/-}$	28.8±3.1***	98.5±9.7*	525.3±48.8***
SIRT6 $^{flox/flox}$	34.0±5.1	116.0±13.8	636.9±78.0
Tie2-Cre; SIRT6 $^{flox/flox}$	36.6±5.2	104.8±8.7	652.3±65.2

Supplementary Table S2. Analysis of progeny from male Tie2-Cre; SIRT6$^{flox/+}$ mice and female SIRT6$^{flox/flox}$ intercross.

Genotypes	SIRT6$^{flox/+}$	SIRT6$^{flox/flox}$	Tie2-Cre; SIRT6$^{flox/+}$	Tie2-Cre; SIRT6$^{flox/flox}$
**Number**	39	32	35	33
**Percentile**	28.1	23.0	25.2	23.7

Supplementary Table S3. Analysis of progeny from male Tie2-Cre; SIRT6$^{flox/+}$ mice and female SIRT6$^{flox/flox}$ intercross.

Lipid profile	Sirt6$^{+/+}$; ApoE$^{-/-}$ (n=7)	Sirt6$^{+/-}$; ApoE$^{-/-}$(n=8)	p Value
TG (mg/dl)	59.89 ± 5.868	59.36 ± 2.140	0.9295
HDL-C (mg/dl)	44.56 ± 9.863	88.60 ± 7.077	0.0027
LDL-C/VLDL-C (mg/dl)	424.1 ± 38.62	352.6 ± 21.72	0.1188

8 weeks Sirt6$^{+/+}$; ApoE$^{-/-}$ and Sirt6$^{+/-}$; ApoE$^{-/-}$ mice were fed a HFD for 8 weeks, then fasted serum was collected for detecting lipid levels as described in Method section.

Supplementary Table S4. Genes significantly down-regulated >40% after the infection with SIRT6 adenovirus for 24 h. Please browse Full text version to see data of this table.

Supplementary Table S5. Genes significantly upregulated by 1-fold after the infection with SIRT6 adenovirus for 24 h. Please browse Full text version to see data of this table.

Supplementary Table S6. List of antibodies.

Antibodies	Supplier, Cat. No.	Dilution	Application
SIRT6	Cell Signaling, #12486	1:1,000	Western blot (mice tissues)
	Abcam, #ab62739	1:1,000	Western blot (mice tissues)
		2 μg/ChIP	ChIP assay
SIRT6	Sigma-Aldrich, #S4322	1:1,000	Western blot
		2 μg/ChIP	ChIP assay
H3K9Ac	Sigma-Aldrich, #H9286	2 μg/ChIP	ChIP assay
ICAM-1	Santa Cruz, #sc-8439	1: 1,000	Western blot
VCAM-1	Santa Cruz, #sc-1504	1: 1,000	Western blot
α-Tubulin	Sigma, #T5168	1:10,000	Western blot
GAPDH	Millipore, #AB2302	1: 5,000	Western blot
Rbt IgG	Santa Cruz, #sc-2027	2 μg/ChIP	ChIP assay

Supplementary Table S7. List of primers
Real-time PCR (qPCR)

Gene	Sequence (5'-3')
mSirt6	Forward: CGTCTGGTCATTGCAACCT<br>Reverse: GAGTCTGCACATCACCTCATC
mGAPDH	Forward: AACAGCAACTCCCACTCTTC<br>Reverse: CCTGTTGCTGTAGCCGTATT
hTNFSF4	Forward: ATCTCCCTGAAGGGCTACTT<br>Reverse:<br>GTTGACAGACCTGACCTTCTTC
hGAPDH	Forward: GATTCCACCCATGGCAAATTC<br>Reverse: CTGGAAGATGGTGATGGGATT

m, mouse; h, human

Supplementary Table S7. List of primers
ChIP-q PCR

Promoter	Sequence (5'-3')
GAPDH*	Forward: AACAGCAACTCCCACTCTTC<br>Reverse: CCTGTTGCTGTAGCCGTATT
TNFSF4	Forward: GGAAGGAAAGGAGACAAGGATAC<br>Reverse: CCCTTTGAGGCAAATTCAATAAAC

*Primer sequence refers to EMD Millipore website (ChIP Validated H3K9Ac Antibody and Primer Set):
http://www.emdmillipore.com/US/en/product/,MM_NF-17-609.
